# 

**Published:** 1898-03

**Authors:** 


### CONTENTS.

ORIGINAL COMMUNICATIONS. PAGE

The Connection between Diseases of the Eye and Diseases of the Teeth. By CHARLES STEDMAN BULL, A.M., M.D., New York . . . . . . . . . . . . . 137

The Place of Local Anæsthesia in Surgery. By F. B. LUND, M.D., Boston, Mass. . . . . . . . . . . . . . . . . . . . . . . . . . . . . . . . 152

Syphilitic Affections of the Mouth. By HENRY BURCHARD, M.D., D.D.S. . . . 162

ABSTRACTS AND TRANSLATIONS.

Dentistry in Foreign Countries . . . . . . . . . . . . . . . . . . . . . . 167

REPORTS OF SOCIETY MEETINGS.

American Academy of Dental Science . . . . . . . . . . . . . . . . . . 176

The New York Institute of Stomatology . . . . . . . . . . . . . . . . . 184

EDITORIAL.

Practice of Dentistry in Foreign Countries . . . . . . . . . . . . . . . . 191

BIBLIOGRAPHY . . . . . . . . . . . . . . . . . . . . . . . . . . . . . . 194

OBITUARY.

Resolutions of Respect—Massachusetts Board of Registration in Dentistry . . . . 195

Resolutions of Respect—American Academy of Dental Science . . . . . . . . . 196

NOTES AND COMMENTS . . . . . . . . . . . . . . . . . . . . . . . . . 197

CURRENT NEWS.

The New York Institute of Stomatology . . . . . . . . . . . . . . . . . 200

Ninth International Congress of Hygiene and Demography . . . . . . . . . . 200

Rhode Island Dental Society . . . . . . . . . . . . . . . . . . . . . . . 200

